# Supplementary material for: Resolving the full spectrum of human genome variation using Linked-Reads
Source: Genome Res. 2019 Apr;29(4):635–45. doi: 10.1101/gr.234443.118 (PMC6442396; doi:10.1101/gr.234443.118)
Supplement: Supplemental Material [file supp_gr.234443.118_Supplemental_longranger-2.2.zip › longranger-2.2.tar.bz2!/lr2.2-public/lib/stan/cmdstan/stan/doc/api/html/structstan_1_1json_1_1json__error.html]

Stan: stan::json::json\_error Struct Reference


|  |  |
| --- | --- |
|  | Stan  2.5.0  probability, sampling & optimization |


- Main Page
- Related Pages
- Namespaces
- Classes
- Files

- Class List
- Class Index
- Class Hierarchy
- Class Members

All Classes Namespaces Files Functions Variables Typedefs Enumerations Enumerator Friends Macros Pages

- stan
- json
- json\_error

Public Member Functions |
List of all members

stan::json::json\_error Struct Reference

Exception type for JSON errors.
More...

`#include <json_error.hpp>`

Inheritance diagram for stan::json::json\_error:

|  |  |
| --- | --- |
| Public Member Functions | |
|  | json\_error (const std::string &what) |
|  | Construct a JSON error with the specified message. More... |
|  | |

## Detailed Description

Exception type for JSON errors.

Definition at line 14 of file json\_error.hpp.

## Constructor & Destructor Documentation

|  |  |  |  |  |  |
| --- | --- | --- | --- | --- | --- |
| stan::json::json\_error::json\_error | ( | const std::string & | *what* | ) |  |

inline

Construct a JSON error with the specified message.

Parameters
:   |  |  |
    | --- | --- |
    | what | Message to attach to error |

Definition at line 19 of file json\_error.hpp.

---

The documentation for this struct was generated from the following file:

- src/stan/io/json/json\_error.hpp


---

[ Stan Home Page ]

*© 2011–2014,
Stan Development Team.*
